# Supplementary material for: Insights into genome recoding from the mechanism of a classic +1-frameshifting tRNA
Source: Nat Commun. 2021 Jan 12;12:328. doi: 10.1038/s41467-020-20373-z (PMC7803779; doi:10.1038/s41467-020-20373-z)
Supplement: Supplementary file 1 — Supplementary Information [file 41467_2020_20373_MOESM1_ESM.pdf]

Supplementary Information

Insights into Genome Recoding from the Mechanism of a Classic +1-Frameshifting tRNA

Howard Gamper[1,5], Haixing Li[2,5], Isao Masuda[1], D. Miklos Robkis[3], Thomas Christian[1], Adam B. Conn[4], Gregor Blaha[4], E. James Petersson[3], Ruben L. Gonzalez, Jr[2,6], and Ya-Ming Hou[1,6]

[1]Department of Biochemistry and Molecular Biology, Thomas Jefferson University, Philadelphia, PA 19107, USA

[2]Department of Chemistry, Columbia University, New York, NY 10027, USA

[3]Department of Chemistry, University of Pennsylvania, Philadelphia, PA 19104, USA

[4]Department of Biochemistry, University of California, Riverside, CA 92521, USA

[5]These authors contributed equally to this work.

[6]Corresponding authors:

rlg2118@columbia.edu (T) 212-854-1096; (F) 212-932-1289

ya-ming.hou@jefferson.edu (T) 215-503-4480; (F) 215-503-4954

Running Title: Mechanism of *SufB2*-induced +1 frameshifting

SUPPLEMENTARY TABLES

Supplementary Table 1. Oligonucleotides used in this work.

Purpose	Direction	Sequence (5'-3')
Primers for constructing the *E. coli* *ProL*-knockout (*ProL*-KO) strain by λ-Red recombination	Forward	CGGTGTGGAAAACGGTAGTATTAGCAGCCACGAGTGTGT AGGCTGGAGCTGCTTC
	Reverse	CCAACCGTAAGGGTTGGTTTTTTCTTGGGATTTTATGGGA ATTAGCCATGGTCC
Primers for constructing the *E. coli* chromosomal *SufB2* strain and the *ProL* counterpart strain by λ-Red recombination	Forward	AACGGTAGTATTAGCAGCCACGAGTCGGCACGTAGCGCA GCCTGGTAGCG
	Reverse-1	GAAGCAGCTCCAGCCTACACGGTTTTTCTTGGGATTTTT GGTCGGCACGAGAGGATTTG
	Reverse-2	GCTGTGATGTGTCACGGTCTGTTTATCGAATTAATTGCAG ATGGGAATTAGCCATGGTCC
Primers for constructing pKK223-3-*SufB2* by Quikchange	Forward	CTGGTAGCGCACCGTCATGGGGGTGTCGGGGGTCGGAG GTTC
	Reverse	GAACCTCCGACCCCCGACACCCCCATGACGGTGCGCTA CCAG
Primers for the CCC-C insertion into the *folA* plasmid for PURExpress by Quikchange	Forward	GATATACATATGATCAGTCTGCCCCATTGCGGCGTTAGC GGTAGATC
	Reverse	GATCTACCGCTAACGCCGCAATGGGGCAGACTGATCATA TGTATATC
Primers for the *in vitro* transcription template for mRNA with CCC-C at the second codon	Forward	AAGCTTAATACGACTCACTATAGGGAAGGAGGTAAAAAT G***CCCC***GTTCTAAGCACCACCACCACCACCACCAC
	Reverse	GTGGTGGTGGTGGTGGTGGTGCTTAGAAC***GGGG***CATTTT TACCTCCTTCCCTATAGTGAGTCGTATTAAGCTT
Primers for the *in vitro* transcription template for the *ProL* tRNA	Forward	AAGCTTAATACGACTCACTATAGGGAGAGCCGCTGATGA GTCCGTGAGGACGAAACGGTACCCGGTACCGTCCGGCA CGTAGCGCAGCCTGGTAG
	Reverse	mUmGGTCGGCACGAGAGGATTTGAACCTCCGACCCCCG ACACCCCATGACGGTGCGCTACCAGGCTG
Primers for the *in vitro* transcription template for the *SufB2* tRNA	Forward	AAGCTTAATACGACTCACTATAGGGAGAGCCGCTGATGA GTCCGTGAGGACGAAACGGTACCCGGTACCGTCCGGCA CGTAGCGCAGCCTGGTAG
	Reverse	mUmGGTCGGCACGAGAGGATTTGAACCTCCGACCCCCG ACACCCCCATGACGGTGCGCTACCAGGCTG
Primers for the *in vitro* transcription template for $tRNA^{Ser/UCA}$	Forward	AAGCTTAATACGACTCACTATAGGTGAGGTGGCCGAGAG GCTGAAGGCGCTCCCCTGCTAAGG
	Reverse	mUmGGCGGTGAGGCGGGGATTCGAACCCCGGATGCAG CTTTTGACCGCATACTCCCTTAGCAGGG
Primer for primer extension inhibition assay for *ProL* and *SufB2*		GATTTGAACCTCCGACCC
Primer for toeprint assay		TCCTTAATTGCCGTTGAGCGAT
3' biotinylated DNA for hybridization with mRNA for smFRET experiments		TGTGTAAGTTTTAGGTTGATTTG-Biotin

Supplementary Table 2. Kinetics of dipeptide and tripeptide formation upon delivery of *SufB2*-TC or *ProL*-TC to the A site of 70S ICs.

**Conversion of fM to fMP ($k_{fMP,obs}$, s^{-1})**			
**TC**	**G37-state**	**m^1G37-state**	**Native-state**
SufB2	0.91 ± 0.09	0.34 ± 0.03	0.49 ± 0.03
ProL	0.83 ± 0.06	1.2 ± 0.1	1.0 ± 0.1
**Conversion of fMP to fMPV ($k_{fMP+V,obs}$, s^{-1})**			
**TC**	**G37-state**	**m^1G37-state**	**Native-state**
SufB2	1.0 ± 0.1	1.7 ± 0.3	2.0 ± 0.4
ProL	6.0 ± 0.7	2.4 ± 0.3	2.3 ± 0.2
**Conversion of fMP to fMPR ($k_{fMP+R,obs}$, s^{-1})**			
**TC**	**G37-state**	**m^1G37-state**	**Native-state**
SufB2	0.6 ± 0.2	0.36 ± 0.03	0.14 ± 0.01
ProL	1.4 ± 0.1	0.9 ± 0.1	1.8 ± 0.1

Supplementary Table 3. Rates and yields of di- and tripeptide formation reactions performed with G37-state *SufB2* in the absence and presence of EF-P.

Reaction	k_{obs} (s^{-1})		Di- or Tripeptide Yield (%)	
	0 μM EF-P	10 μM EF-P	0 μM EF-P	10 μM EF-P
Conversion of fM to fMP	0.91 ± 0.09	1.2 ± 0.1	50	50
Conversion of fM to fMPV	0.06 ± 0.01	0.11 ± 0.01	58	53
Conversion of fM to fMPR	0.04 ± 0.01	0.07 ± 0.01	8.2	4.9
Conversion of fMP to fMPV	1.0 ± 0.1	1.00 ± 0.07	53	53
Conversion of fMP to fMPR	0.6 ± 0.1	0.6 ± 0.1	8.8	8.7

Supplementary Table 4. Rate constants characterizing the initial 70S IC→GS2 transition and rate constants and equilibrium constants characterizing the subsequent GS1⇄GS2 equilibrium that are observed upon delivery of *SufB2-* or *ProL*-TC to the A site of 70S ICs in the absence of EF-G.

Pre-steady-state movie[a]

Components	$k_{70S\ IC\rightarrow GS2}$ (s^{-1})	$k_{GS1\rightarrow GS2}$ (s^{-1})	$k_{GS2\rightarrow GS1}$ (s^{-1})	K_{eq}
SufB2-TC	0.30 ± 0.04	0.58 ± 0.02	0.32 ± 0.01	1.81 ± 0.08
ProL-TC	0.6 ± 0.2	0.82 ± 0.04	0.45 ± 0.02	1.82 ± 0.12

Steady-state movie at 1 min post-delivery[a]

Components	$k_{70S\ IC\rightarrow GS2}$ (s^{-1})	$k_{GS1\rightarrow GS2}$ (s^{-1})	$k_{GS2\rightarrow GS1}$ (s^{-1})	K_{eq}
SufB2-TC	N.A.[b]	0.232 ± 0.006	0.236 ± 0.006	0.98 ± 0.04
ProL-TC	N.A.	0.333 ± 0.009	0.32 ± 0.01	0.96 ± 0.04

[a] The small differences between the rate constants and equilibrium constants obtained from the analysis of the pre-steady-state movie and those obtained from the analysis of the steady-state movie most likely arise from the fact that the pre-steady-state movies capture PRE complexes that have not yet reached full conformational equilibrium. For this reason, the rate constants and equilibrium constants obtained from the analyses of the steady-state movies are the ones that are reported in the text of the article.

[b] N.A. refers to values that are not applicable.

Supplementary Table 5. Rate constants characterizing the initial 70S IC→GS2 transition and rate constants and fractional populations characterizing the GS2→POST transition that are observed upon delivery of *SufB2*- or *ProL*-TC to the A site of 70S ICs in the presence of EF-G.

Pre-steady-state movie

Components	$k_{70S\ IC \rightarrow GS2}$ (s^{-1})	$k_{GS2 \rightarrow POST}$ (s^{-1})[a]	POST (%)
SufB2-TC + EF-G	0.33 ± 0.05	N.A.	20.4 ± 0.2
ProL-TC + EF-G	0.31 ± 0.05	0.35 ± 0.02	42.8 ± 0.3

Steady-state movie at 1 min post-delivery

Components	$k_{70S\ IC \rightarrow GS2}$ (s^{-1})	$k_{GS2 \rightarrow POST}$ (s^{-1})[a]	POST (%)
SufB2-TC + EF-G	N.A.	N.A.	35.5 ± 0.1
ProL-TC + EF-G	N.A.	N.A.	72.6 ± 0.1

Steady-state movie at 3 min post-delivery

Components	$k_{70S\ IC \rightarrow GS2}$ (s^{-1})	$k_{GS2 \rightarrow POST}$ (s^{-1})[a]	POST (%)
SufB2-TC + EF-G	N.A.	N.A.	34.6 ± 0.1

Steady-state movie at 10 min post-delivery

Components	$k_{70S\ IC \rightarrow GS2}$ (s^{-1})	$k_{GS2 \rightarrow POST}$ (s^{-1})[a]	POST (%)
SufB2-TC + EF-G	N.A.	N.A.	56.2 ± 0.1

Steady-state movie at 20 min post-delivery

Components	$k_{70S\ IC \rightarrow GS2}$ (s^{-1})	$k_{GS2 \rightarrow POST}$ (s^{-1})[a]	POST (%)
SufB2-TC + EF-G	N.A.	N.A.	63.4 ± 0.1

[a] N.A. refers to values that were not applicable.

Supplementary Table 6. Rate constants and equilibrium constants characterizing the GS1⇄GS2 equilibrium that is observed in a sub-population of PRE complexes that lack an A site-bound, deacylated *SufB2* at the longer time points (i.e., 3, 10, and 20 min) post-delivery of *SufB2*-TC to the A site of 70S ICs in the presence of EF-G.

Steady-state movie at 3 min post-delivery

Components	$k_{GS1 \rightarrow GS2}$ (s^{-1})	$k_{GS2 \rightarrow GS1}$ (s^{-1})	K_{eq}
SufB2-TC + EF-G	0.260 ± 0.006	0.130 ± 0.003	2.00 ± 0.07

Steady-state movie at 10 min post-delivery

Components	$k_{GS1 \rightarrow GS2}$ (s^{-1})	$k_{GS2 \rightarrow GS1}$ (s^{-1})	K_{eq}
SufB2-TC + EF-G	0.208 ± 0.004	0.245 ± 0.005	0.85 ± 0.02

Steady-state movie at 20 min post-delivery

Components	$k_{GS1 \rightarrow GS2}$ (s^{-1})	$k_{GS2 \rightarrow GS1}$ (s^{-1})	K_{eq}
SufB2-TC + EF-G	0.180 ± 0.003	0.301 ± 0.006	0.60 ± 0.02

Supplementary Table 7. Rate constants and equilibrium constants characterizing the GS1⇄GS2 equilibrium of the *SufB2*- and *ProL*-PRE^{-A} complexes.

Steady-state movie			
**PRE^{-A} Complex**	**$k_{GS1\rightarrow GS2}$ (s^{-1})[a]**	**$k_{GS2\rightarrow GS1}$ (s^{-1})[a]**	**K_{eq}[a]**
SufB2	0.227 ± 0.002	0.363 ± 0.003	0.625 ± 0.008
ProL	0.412 ± 0.003	0.267 ± 0.002	1.54 ± 0.02

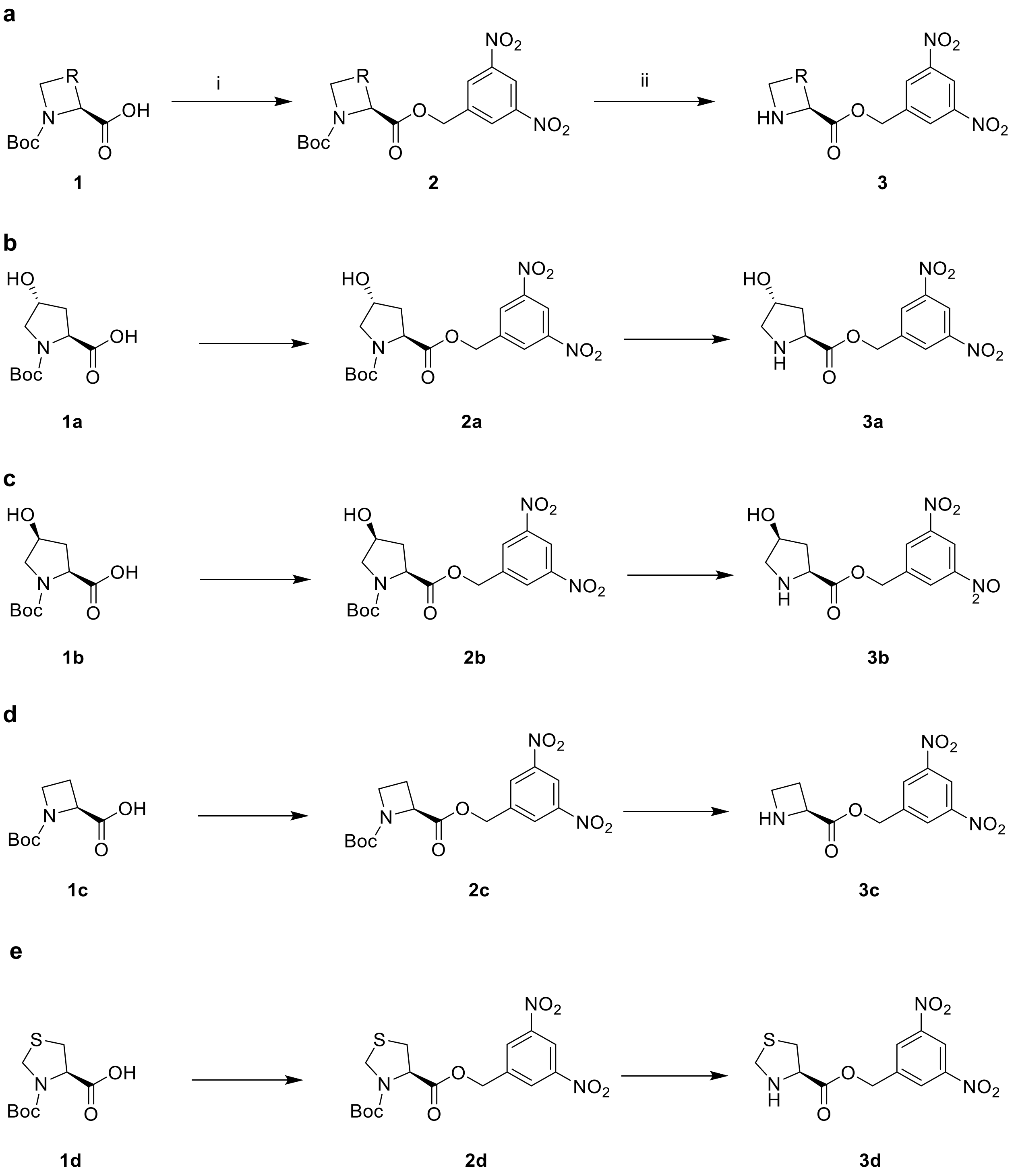

Supplementary Figure 1. a Syntheses of proline dinitrobenzyl ester analogs. The generalized synthesis scheme that was used for generating the proline dinitrobenzyl ester analogs. Step *i* was performed in 3,5-dinitrobenzyol chloride, sodium iodide (NaI), N,N-diisopropylethylamine

(DIPEA), and tetrahydrofuran (THF) and step *ii* was performed in trifluoroacetic acid (TFA) and trichloromethane ($CHCl_3$). All chemicals were purchased from Sigma-Aldrich (St. Louis, MO) or Fisher Scientific (Waltham, MA). Boc-protected proline derivatives were purchased from ChemImpex (Wood Dale, IL). All reagents were used without further purification. Preparative high-performance LC (HPLC) was performed on a Varian Prostar HPLC system (Agilent Technologies, Santa Clara, CA) using a gradient elution from 0:100 acetonitrile:water with 0.1% TFA to 45:55 acetonitrile:water with 0.1% TFA. A Waters Sunfire C18 OBD Prep column was used to purify all final products. **b** Synthesis of trans-L-4-hydroxyproline dinitrobenzyl ester (3a). Boc-trans-L-4-hydroxyproline (1a) (231 mg, 1.00 mmol), 3,5-dinitrobenzoyl chloride (543 mg, 2.5 mmol), and NaI (450 mg, 3.00 mmol) were dissolved in THF (10 mL). DIPEA (695 µL, 4.00 mmol) was added and the reaction was stirred at room temperature overnight. The next day, the crude reaction mixture was partitioned between 100 mL water (H_2O)/ethyl acetate (EtOAc) (1:1). The organic phase was washed twice with a saturated ammonium chloride (NH_4Cl) solution and once with brine before being dried over magnesium sulfate ($MgSO_4$). The solution was then filtered and the solvent was removed *in vacuo*. Crude product was dissolved in dichloromethane (CH_2Cl_2) and purified over silica using 5% methanol (MeOH) in CH_2Cl_2 (R_f = 0.3). Pure fractions were combined and the solvent was removed *in vacuo*. Purified Boc-trans-L-4-hydroxyproline dinitrobenzyl ester (2a) was produced in 46.0% yield as an orange foam. 2a (95 mg, 0.23 mmol) was dissolved in $CHCl_3$ (1mL), and TFA (1mL) was added while stirring. After 1 hour stirring at room temperature, the solvent was removed *in vacuo*. The crude product was dissolved in 50% acetonitrile (MeCN) in H_2O and purified by reverse phase HPLC. The desired product, 3a, eluted with approximately 24% MeCN and was isolated in high purity. After lyophilization, 3a was dissolved in dimethyl sulfoxide (DMSO) to yield a 25 mM stock solution used in later experiments. **c** Synthesis of trans-L-4-hydroxyproline dinitrobenzyl ester (3b). Boc-cis-L-4-hydroxyproline (1b) (231 mg, 1.00 mmol), 3,5-dinitrobenzyol chloride (543 mg, 2.5 mmol), and NaI (450 mg, 3.00 mmol) were dissolved in THF (10 mL). DIPEA (695 µL, 4.00 mmol) was added and the reaction was stirred at room

temperature overnight. The next day, the crude reaction mixture was partitioned between 100 mL H_2O/EtOAc (1:1). The organic phase was washed twice with a saturated NH_4Cl solution and once with brine before being dried over $MgSO_4$. The solution was then filtered and the solvent was removed *in vacuo*. Crude product was dissolved in CH_2Cl_2 and purified over silica using 5% MeOH in CH_2Cl_2 (R_f = 0.3). Pure fractions were combined and the solvent was removed *in vacuo*. Purified Boc-cis-L-4-hydroxyproline dinitrobenzyl ester (2b) was produced in 30.4% yield as an orange foam. 2b (62 mg, 0.15 mmol) was dissolved in $CHCl_3$ (1mL), and TFA (1mL) was added while stirring. After 1 hour stirring at room temperature, the solvent was removed *in vacuo*. The crude product was dissolved in 50% MeCN in H_2O and purified by reverse phase HPLC. The desired product, 3b, eluted with approximately 26% MeCN and was isolated in high purity. After lyophilization, 3b was dissolved in DMSO to yield a 25 mM stock solution used in later experiments. **d** Synthesis of 2-carboxyazetidine dinitrobenzyl ester (3c). Boc-L-azetidine-2-carboxylic acid (1c) (201 mg, 1.00 mmol), 3,5-dinitrobenzoyl chloride (543 mg, 2.5 mmol), and NaI (450 mg, 3.00 mmol) were dissolved in THF (10 mL). DIPEA (695 µL, 4.00 mmol) was added and the reaction was stirred at room temperature overnight. The next day, the crude reaction mixture was partitioned between 100 mL H_2O/EtOAc (1:1). The organic phase was washed twice with a saturated NH_4Cl solution and once with brine before being dried over $MgSO_4$. The solution was then filtered and the solvent was removed *in vacuo*. Crude product was dissolved in CH_2Cl_2 and purified over silica using 2% MeOH in CH_2Cl_2 (R_f = 0.3). Pure fractions were combined and the solvent was removed *in vacuo*. Purified Boc-L-azetidine-2-carboxylic acid dinitrobenzyl ester, 2c, was produced in 70.3% yield as a red oil. 2c (134 mg, 0.35 mmol) was dissolved in $CHCl_3$ (1mL), and TFA (1mL) was added while stirring. After 1 hour stirring at room temperature, the solvent was removed *in vacuo*. The crude product was dissolved in 50% MeCN in H_2O and purified by reverse phase HPLC. The desired product, 3c, eluted with approximately 27% MeCN and was isolated in high purity. After lyophilization, 3c was dissolved in DMSO to yield a 25 mM stock solution used in later experiments. **e** Synthesis of thiaproline dinitrobenzyl ester (3d). Boc-

L-thiaproline (1d) (116 mg, 0.50 mmol) and 3,5-dinitrobenzyol chloride (217 mg, 1.0 mmol) were dissolved in THF (5 mL). DIPEA (348 μL, 2.00 mmol) was added and the reaction was stirred at room temperature overnight. The next day, the crude reaction mixture was partitioned between 100 mL H_2O/EtOAc (1:1). The organic phase was washed twice with a saturated NH_4Cl solution and once with brine before being dried over $MgSO_4$. The solution was then filtered and the solvent was removed *in vacuo*. Crude product was dissolved in CH_2Cl_2 and purified over silica using 1% MeOH in CH_2Cl_2 (R_f = 0.4). Pure fractions were combined and the solvent was removed *in vacuo*. Purified Boc-L-thiaproline dinitrobenzyl ester (2d) was produced in 77.5% yield as a yellow oil. 2d (160 mg, 0.39 mmol) was dissolved in $CHCl_3$ (1mL), and TFA (1mL) was added while stirring. After 1 hour stirring at room temperature, the solvent was removed *in vacuo*. The crude product was dissolved in 50% MeCN in H_2O and purified by reverse phase HPLC. The desired product, 3d, eluted with approximately 30% MeCN and was isolated in high purity. After lyophilization, 3d was dissolved in DMSO to yield a 25 mM stock solution used in later experiments.

Nuclear magnetic resonance (NMR) and electrospray ionization mass spectrometry (ESI-MS) characterization of proline dinitrobenzyl ester analogs

NMR spectra were obtained on a Bruker DRX 500 MHz instrument (Billerica, MA). Electrospray ionization high-resolution mass spectrometry (ESI-HRMS) spectra were collected with a Waters LCT Premier XE liquid chromatograph-mass spectrometer (LC-MS) (Milford, MA). Electrospray ionization low-resolution mass spectrometry (ESI-LRMS) spectra were obtained on a Waters Acquity Ultra Performance LC connected to a single-quadrupole-detector (SQD) MS.

2a: ^{1}H NMR (500 MHz, $CDCl_3$-d) δ 8.93 (dt, J = 18.1, 2.1 Hz, 1H), 8.54 (dd, J = 14.5, 2.1 Hz, 2H), 5.39 – 5.24 (m, 2H), 4.51 – 4.42 (m, 2H), 3.56 (ddd, J = 15.8, 11.6, 4.2 Hz, 1H), 3.47 (ddt, J = 34.5, 11.6, 1.8 Hz, 1H), 2.38 – 2.24 (m, 1H), 2.08 – 1.98 (m, 1H), 1.33 (d, J = 38.8 Hz, 9H). ^{13}C NMR (126 MHz, $CDCl_3$) δ 172.65, 154.70, 153.97, 148.63, 140.43, 140.06, 127.89, 118.52, 80.72,

70.03, 69.12, 64.31, 57.76, 54.79, 39.09, 38.44, 28.24. ESI$^+$-HRMS: Calculated for $C_{17}H_{21}N_3O_9Na^+$: 434.1175; Found [M + Na]$^+$: 434.1171.

2b: ^{1}H NMR (500 MHz, $CDCl_3$-d) δ 8.96 (dt, J = 10.0, 2.1 Hz, 1H), 8.59 (dd, J = 9.2, 2.1 Hz, 2H), 5.43 (t, J = 13.4 Hz, 1H), 5.34 (dd, J = 13.7, 7.3 Hz, 1H), 4.52 – 4.44 (m, 1H), 4.44 – 4.39 (m, 1H), 3.61 – 3.51 (m, 2H), 3.03 (s, 1H), 2.42 – 2.30 (m, 1H), 2.23 – 2.13 (m, 1H), 1.39 (d, J = 35.2 Hz, 9H). ^{13}C NMR (126 MHz, $CDCl_3$) δ 174.04, 154.62, 148.69, 140.36, 127.80, 118.46, 80.70, 71.05, 69.90, 64.67, 57.78, 55.73, 38.04, 28.36. ESI$^+$-HRMS: Calculated for $C_{17}H_{21}N_3O_9Na^+$: 434.1175; Found [M + Na]$^+$: 434.1205.

2c: ^{1}H NMR (500 MHz, $CDCl_3$-d) δ 8.88 (s, 1H), 8.53 (d, J = 2.1 Hz, 2H), 5.36 (d, J = 2.8 Hz, 2H), 4.67 (dd, J = 9.2, 5.4 Hz, 1H), 3.91 (dtd, J = 45.7, 8.4, 5.9 Hz, 2H), 2.59 – 2.41 (m, 1H), 2.25 – 2.08 (m, 1H), 1.30 (s, 9H). ^{13}C NMR (126 MHz, $CDCl_3$) δ 170.85, 148.43, 140.19, 127.78, 118.37, 80.19, 64.21, 59.64, 48.03, 28.36, 20.11. ESI$^+$-HRMS: Calculated for $C_{16}H_{21}N_3O_8Na^+$: 404.1070; Found [M + Na]$^+$: 404.1086

2d: ^{1}H NMR (500 MHz, $CDCl_3$-d) δ 8.92 (d, J = 6.6 Hz, 1H), 8.53 (d, J = 2.1 Hz, 2H), 5.47 – 5.28 (m, 2H), 4.93 – 4.76 (m, 1H), 4.56 (dd, J = 22.7, 8.9 Hz, 1H), 4.42 (t, J = 8.0 Hz, 1H), 3.40 – 3.26 (m, 1H), 3.22 – 3.13 (m, 1H), 1.37 (d, J = 36.1 Hz, 9H). ^{13}C NMR (126 MHz, $CDCl_3$) δ 170.23, 153.23, 148.61, 140.18, 127.60, 118.44, 81.48, 64.63, 61.65, 48.32, 33.31, 28.16. ESI$^+$-HRMS: Calculated for $C_{16}H_{19}N_3O_8SNa^+$: 436.0791; Found [M + Na]$^+$: 436.0796 .

3a: ^{1}H NMR (500 MHz, DMSO-d_6) δ 9.75 (s, 2H), 8.82 (t, J = 2.1 Hz, 1H), 8.75 (d, J = 2.1 Hz, 2H), 5.60 (s, 1H), 5.49 (s, 2H), 4.66 (dd, J = 10.7, 7.6 Hz, 1H), 4.48 – 4.44 (m, 1H), 3.38 (dd, J = 12.1, 4.2 Hz, 1H), 3.13 (dt, J = 12.1, 1.6 Hz, 1H), 2.30 – 2.14 (m, 2H). ^{13}C NMR (126 MHz, DMSO-d_6)

δ 168.50, 148.09, 139.56, 128.56, 118.39, 68.48, 65.17, 57.62, 53.40, 36.93. ESI^+-HRMS: Calculated for $C_{12}H_{14}N_3O_7^+$: 312.0832; Found $[M + H]^+$: 312.0833.

3b: ^1H NMR (500 MHz, DMSO-d_6) δ 9.74 (s, 2H), 8.81 (t, *J* = 2.1 Hz, 1H), 8.74 (d, *J* = 2.1 Hz, 2H), 5.51 (d, *J* = 2.1 Hz, 2H), 4.71 (dd, *J* = 9.7, 3.4 Hz, 1H), 4.40 (dt, *J* = 4.2, 2.1 Hz, 1H), 3.73 (s, 1H), 3.28 (dd, *J* = 11.9, 4.0 Hz, 1H), 3.21 (dt, *J* = 11.5, 1.5 Hz, 1H), 2.36 (ddd, *J* = 13.8, 9.7, 4.3 Hz, 1H), 2.24 (ddt, *J* = 13.4, 3.4, 1.8 Hz, 1H). ^{13}C NMR (126 MHz, DMSO-d_6) δ 169.07, 148.07, 139.70, 128.53, 118.33, 68.17, 65.20, 57.70, 53.28, 37.15. ESI^+-HRMS: Calculated for $C_{12}H_{13}N_3O_7Na^+$: 334.0651; Found $[M + Na]^+$: 334.0673.

3c: ^1H NMR (500 MHz, DMSO-d_6) δ 8.82 (t, J = 2.2 Hz, 1H), 8.75 (d, J = 2.1 Hz, 2H), 5.50 (s, 2H), 5.28 (t, J = 9.0 Hz, 1H), 4.01 (dt, J = 10.0, 8.8 Hz, 1H), 3.81 (ddd, J = 10.0, 8.6, 7.1 Hz, 2H), 2.73 – 2.64 (m, 2H). ^{13}C NMR (126 MHz, DMSO) δ 167.86, 148.10, 139.51, 128.73, 118.44, 65.12, 56.54, 43.02, 22.49. ESI^+-HRMS: Calculated for $C_{11}H_{12}N_3O_6^+$: 282.0726; Found $[M + H]^+$: 282.0728.

3d: ^1H NMR (500 MHz, DMSO-d_6) δ 8.80 (t, *J* = 2.1 Hz, 1H), 8.70 (d, *J* = 2.1 Hz, 2H), 5.43 (d, *J* = 4.5 Hz, 2H), 4.31 (dd, *J* = 7.1, 5.7 Hz, 1H), 4.17 (q, *J* = 9.1 Hz, 2H), 3.13 (dd, *J* = 10.5, 7.1 Hz, 1H), 3.03 (dd, *J* = 10.4, 5.7 Hz, 1H). ^{13}C NMR (126 MHz, DMSO) δ 170.32, 148.08, 140.22, 128.18, 118.19, 64.36, 64.27, 53.30, 35.21. ESI^+-HRMS: Calculated for $C_{11}H_{12}N_3O_6S^+$: 314.0447; Found $[M + H]^+$: 314.0443.

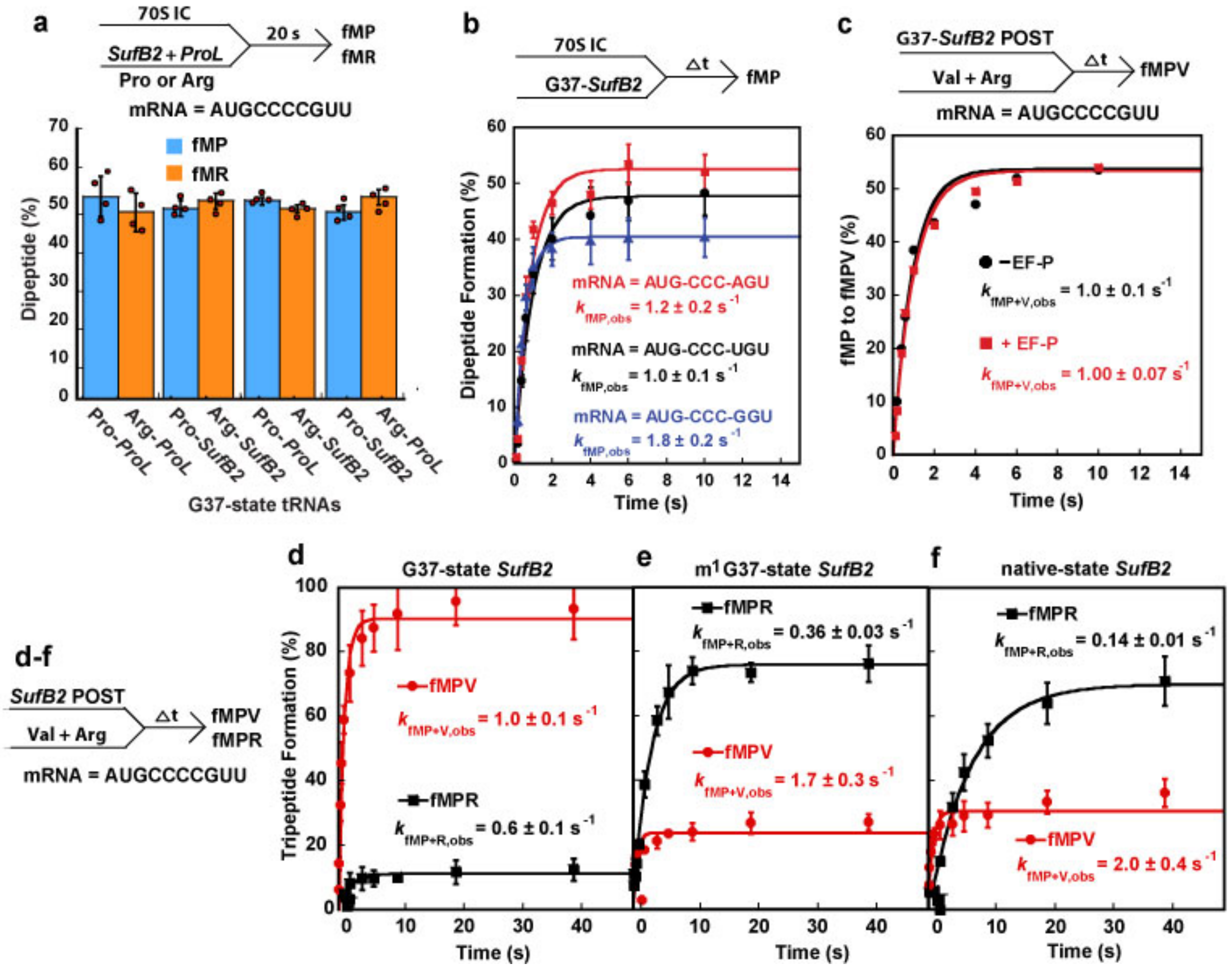

Supplementary Figure 2. Dipeptide and tripeptide formation assays. **a** Dipeptide formation assays comparing the yields of G37-state *SufB2*- and *ProL*-TC. Each tRNA was pre-aminoacylated with Pro or Arg, as indicated on the X axis of the bar graph, and used to form TCs. Equimolar mixtures of the pairs of TCs indicated on the X axis of the bar graph were then delivered to the A site of a 70S IC. **b** Plot of fMP formation as a function of time for delivery of G37-state *SufB2*-TC to the A site of 70S ICs programmed with a CCC-N motif at the A site in which N = A, U, or G. **c** Relative fMPV and fMPR formation as a function of time upon rapid delivery of an equimolar mixture of $tRNA^{Arg}$- and $tRNA^{Val}$-TCs to POST complexes carrying G37-state fMP-*SufB2* at the P site in the absence or presence of 10 μM β-lysinylated EF-P. The mRNA coding sequence is AUG-CCC-CGU-U. **d-f** Relative fMPV and fMPR formation as a function of time upon

rapid delivery of an equimolar mixture of $tRNA^{Arg}$- and $tRNA^{Val}$-TCs to POST complexes carrying **d** G37-state-, **e** m^1G37-state-, or **f** native-state fMP-*SufB2* at the P site. The mRNA coding sequence is AUG-CCC-CGU-U. These kinetic and yield plots correspond to the bar graphs in Figure 5c. In panel **a**, the bars are SD of four independent experiments (n = 4), whereas in panels **b**-**f**, the bars are SD of three independent experiments (n = 3). All data are presented as mean values ± SD. Δt: a time interval.

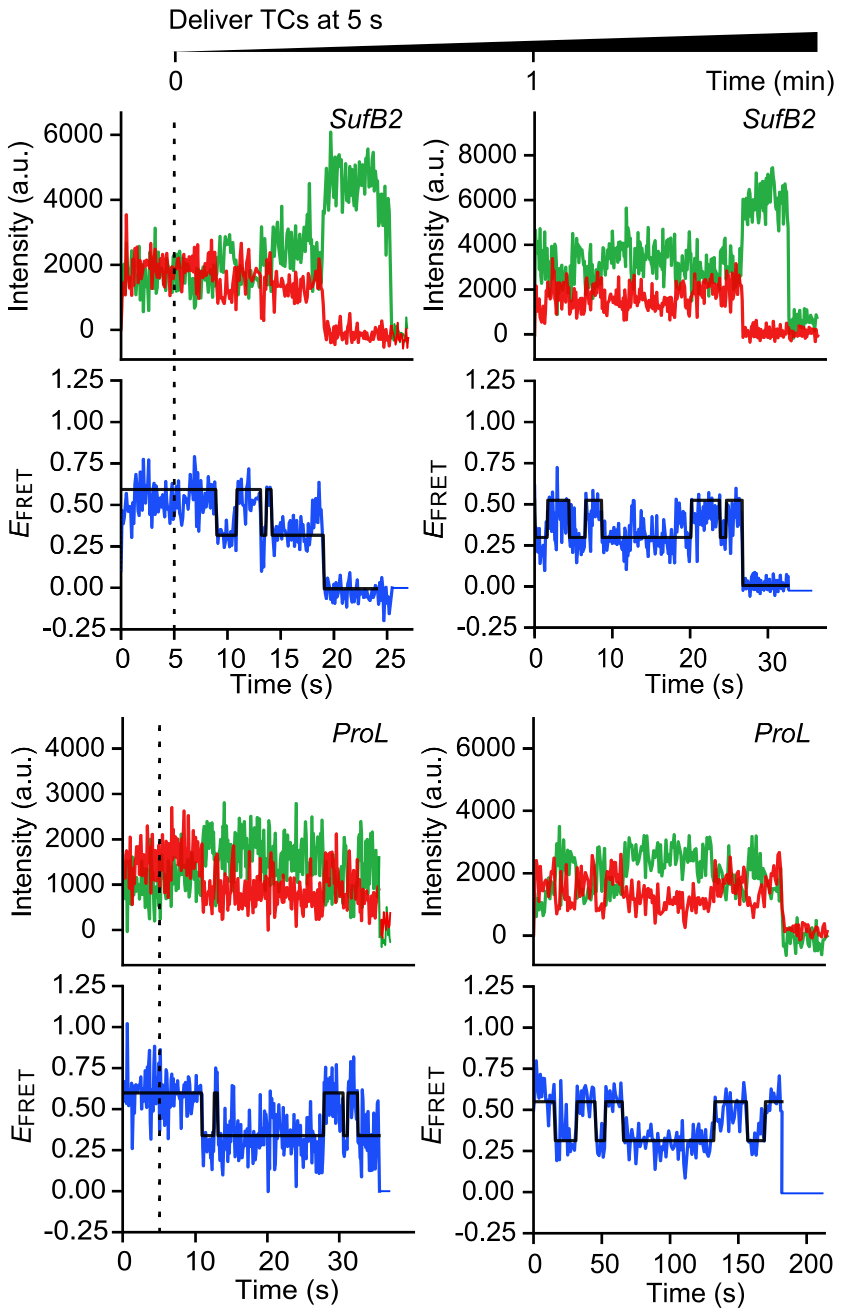

Supplementary Figure 3. Representative fluorescence intensity and E_{FRET} vs. time trajectories recorded in the absence of EF-G. Representative Cy3 (green) and Cy5 (red) fluorescence intensity vs. time trajectories and corresponding E_{FRET} (blue) vs. time trajectories for ribosomal complexes described in Figures 6**a**, 6**d**, and 6**f**. The Viterbi paths (black) are superimposed on the E_{FRET} trajectories. The black vertical dashed lines in the leftmost plots indicate the time at which the TCs were stopped-flow delivered.

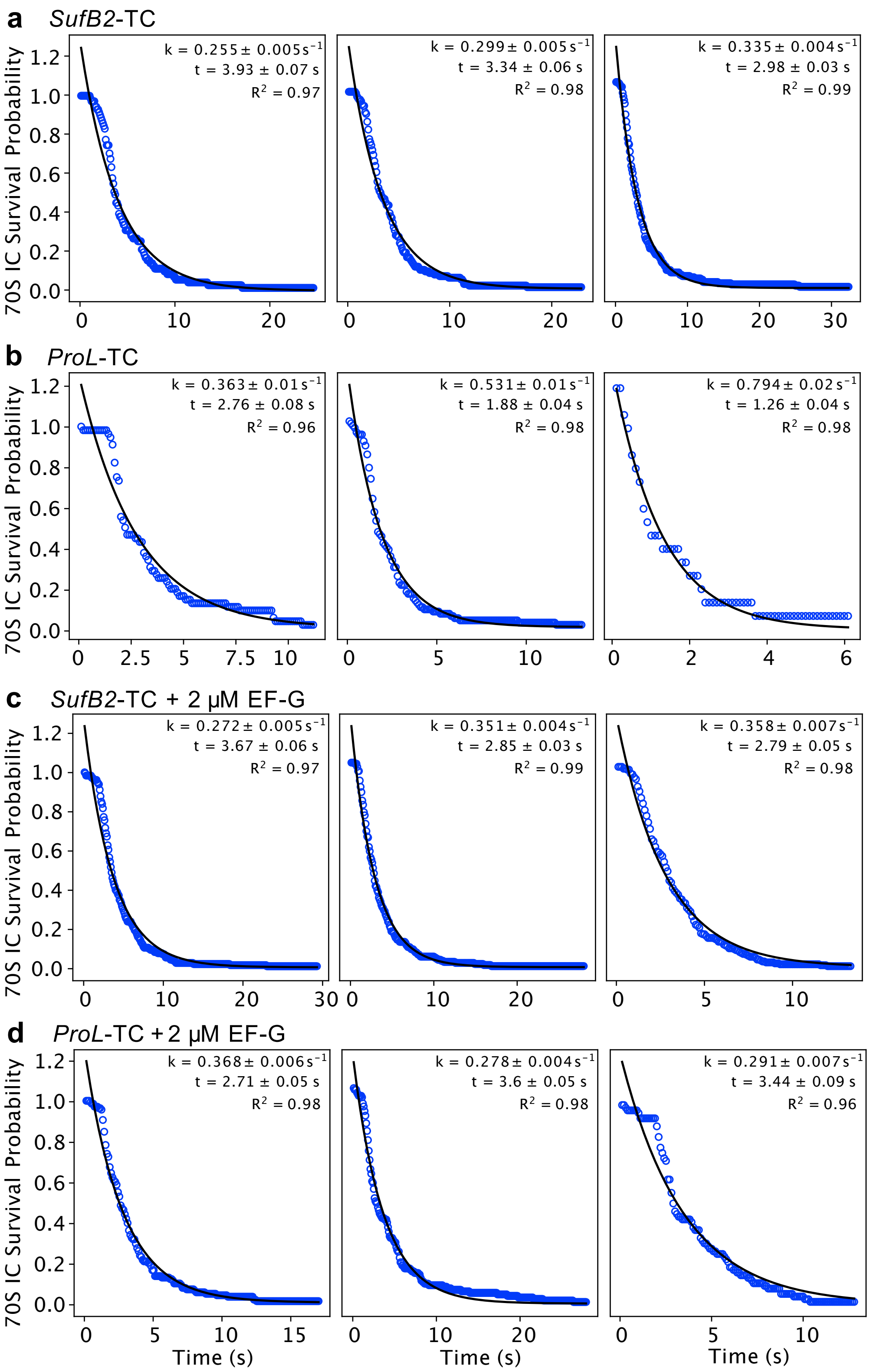

Supplementary Figure 4. Plots of the 70S IC survival probability vs. time used to determine $k_{70S\ IC \to GS2}$**. a**, **c** *SufB2*-TC or **b**, **d** *ProL*-TC in the **a**, **b** absence or **c**, **d** presence of EF-G were

stopped-flow delivered to 70S ICs and the survival probability of the 70S IC prior to undergoing a transition to GS2 (blue circles) was plotted as a function of time. The resulting survival probability plots were well described by a single exponential decay (black solid line), which was used to determine $k_{70S\ IC \rightarrow GS2}$, as described in the *Analysis of smFRET experiments* section of the Methods. Three technical replicates of each experiment were performed and the survival probability plot of each replicate is shown. The mean value and standard deviation of the $k_{70S\ IC \rightarrow GS2}$ for each condition reported in Supplemental Tables 4 and 5 was determined from the analysis of the three technical replicates as described in the Analysis of smFRET experiments section of Methods.

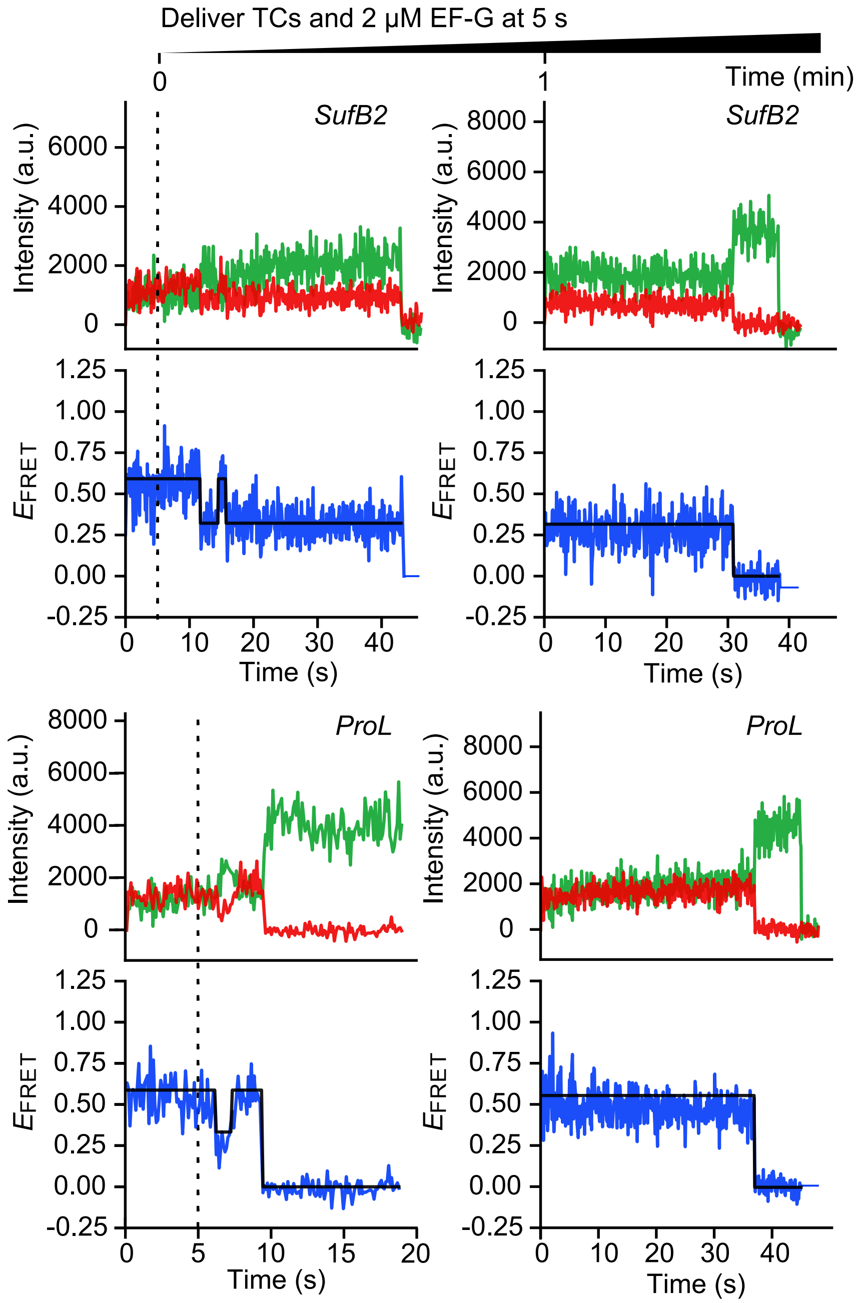

Supplementary Figure 5. Representative fluorescence intensity and E_{FRET} vs. time trajectories recorded in the presence of EF-G. Representative Cy3 (green) and Cy5 (red) fluorescence intensity vs. time trajectories and corresponding E_{FRET} (blue) vs. time trajectories for ribosomal complexes described in Figures 6**b**, 6**e**, and 6**g**. The Viterbi paths (black) are superimposed on the E_{FRET} trajectories. The black vertical dashed lines in the leftmost plots indicate the time at which the mixture of TC and EF-G was stopped-flow delivered.

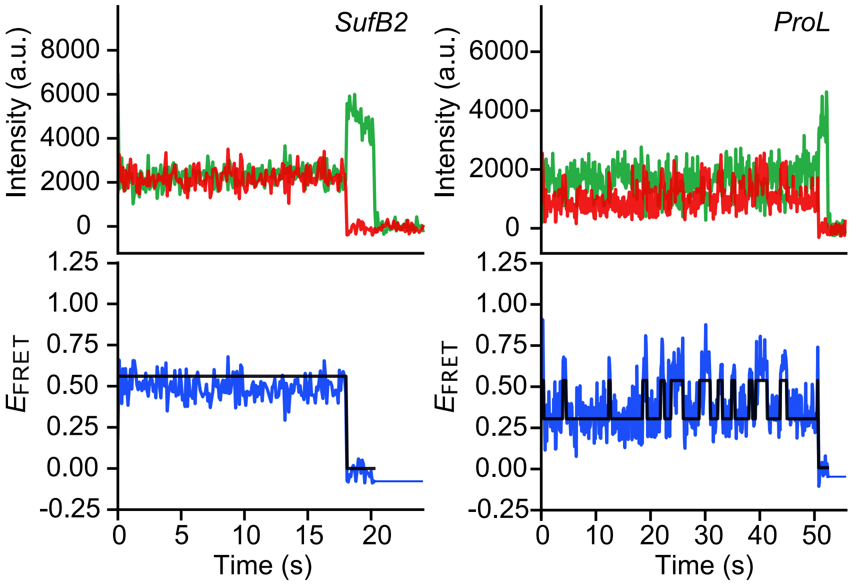

Supplementary Figure 6. Representative fluorescence intensity and E_{FRET} vs. time trajectories recorded for PRE^{-A} complexes. Representative Cy3 (green) and Cy5 (red) fluorescence intensity vs. time trajectories and corresponding E_{FRET} (blue) vs. time trajectories for ribosomal complexes described in Figures 6**c** and 6**h**. The Viterbi paths (black) are superimposed on the E_{FRET} trajectories.
