# Supplementary material for: Insights into genome recoding from the mechanism of a classic +1-frameshifting tRNA
Source: Nat Commun. 2021 Jan 12;12:328. doi: 10.1038/s41467-020-20373-z (PMC7803779; doi:10.1038/s41467-020-20373-z)
Supplement: Supplementary file 2 — Reporting Summary [file 41467_2020_20373_MOESM2_ESM.pdf]

nature research

Corresponding author(s): Ruben L. Gonzalez Jr., Ya-Ming Hou

Last updated by author(s): Nov 10, 2020

Reporting Summary

Nature Research wishes to improve the reproducibility of the work that we publish. This form provides structure for consistency and transparency in reporting. For further information on Nature Research policies, see our Editorial Policies and the Editorial Policy Checklist.

Statistics

For all statistical analyses, confirm that the following items are present in the figure legend, table legend, main text, or Methods section.

n/a	Confirmed	
☐	☒	The exact sample size (*n*) for each experimental group/condition, given as a discrete number and unit of measurement
☐	☒	A statement on whether measurements were taken from distinct samples or whether the same sample was measured repeatedly
☒	☐	The statistical test(s) used AND whether they are one- or two-sided<br>*Only common tests should be described solely by name; describe more complex techniques in the Methods section.*
☐	☒	A description of all covariates tested
☐	☒	A description of any assumptions or corrections, such as tests of normality and adjustment for multiple comparisons
☐	☒	A full description of the statistical parameters including central tendency (e.g. means) or other basic estimates (e.g. regression coefficient) AND variation (e.g. standard deviation) or associated estimates of uncertainty (e.g. confidence intervals)
☒	☐	For null hypothesis testing, the test statistic (e.g. *F*, *t*, *r*) with confidence intervals, effect sizes, degrees of freedom and *P* value noted<br>*Give P values as exact values whenever suitable.*
☐	☒	For Bayesian analysis, information on the choice of priors and Markov chain Monte Carlo settings
☒	☐	For hierarchical and complex designs, identification of the appropriate level for tests and full reporting of outcomes
☒	☐	Estimates of effect sizes (e.g. Cohen's *d*, Pearson's *r*), indicating how they were calculated

Our web collection on statistics for biologists contains articles on many of the points above.

Software and code

Policy information about availability of computer code

Data collection	µManager 1.4 (https://micro-manager.org/) for the collection of TIRF movies.
Data analysis	Image Lab software (Bio-Rad) for PC version 6.1 SOFT-LIT-170-9690-ILSPC-V-6-1; ImageQuant (Cytiva) version TL8.2; Kaleidagraph version 4.5.4; Image J (NIH) version 64-bit Java 1.8.0_172. The code (including Viterbi algorithm) used to analyze the TIRF movies in this study is associated with a manuscript in preparation (Jason Hon, Colin Kinz-Thompson, and Ruben L. Gonzalez), where R.L.G. will be the corresponding author. Consequently, the code will be available from R.L.G., a co-corresponding author of this manuscript, upon request.

For manuscripts utilizing custom algorithms or software that are central to the research but not yet described in published literature, software must be made available to editors and reviewers. We strongly encourage code deposition in a community repository (e.g. GitHub). See the Nature Research guidelines for submitting code & software for further information.

Data

Policy information about availability of data

All manuscripts must include a data availability statement. This statement should provide the following information, where applicable:

- Accession codes, unique identifiers, or web links for publicly available datasets
- A list of figures that have associated raw data
- A description of any restrictions on data availability

Figures that have associated raw data are Figures 2A, 2B, 3C, 3D, 5C, Supplementary Figure 1A, and Supplementary Tables 2, 3, 4, 5, 6, and 7. Due to the lack of a public repository for smFRET data, the smFRET data supporting the findings of this study are available from the corresponding author (R.L.G) upon request.

Field-specific reporting

Please select the one below that is the best fit for your research. If you are not sure, read the appropriate sections before making your selection.

- [x] Life sciences
- [ ] Behavioural & social sciences
- [ ] Ecological, evolutionary & environmental sciences

For a reference copy of the document with all sections, see nature.com/documents/nr-reporting-summary-flat.pdf

Life sciences study design

All studies must disclose on these points even when the disclosure is negative.

Sample size	For biological and enzyme-based in vitro assays, a sample size of 3 was chosen following previously established work to evaluate the standard deviation (SD). If the SD is more than 10% of individual experiments, the sample size would be increased to 5-6, until the SD value drops down to below 10%.
Data exclusions	As described in the Methods section of our manuscript, EFRET vs. time trajectories were excluded from further analysis if transitions in the corresponding Cy3 and Cy5 fluorescence intensity vs. time trajectories were not anti-correlated or if the Cy3 fluorescence intensity vs. time trajectory did not undergo single-step Cy3 photobleaching. These criteria are well-established and standard in the smFRET field. As also described in the Methods section of our manuscript, EFRET vs. time trajectories that were extracted from pre-steady-state movies and that did not meet two additional criteria were also excluded from further analysis: (i) those that did not stably sample EFRET = 0.55 prior to EF-Tu (GTP)aa-tRNA ternary complex (TC) delivery and (ii) those that did not exhibit at least one 0.55→0.31 transition after TC delivery. Applying these two criteria to the EFRET vs. time trajectories extracted from pre-steady-state movies ensured that only bone fide ribosomal 70S initiation complexes (ICs) that underwent TC delivery and peptide-bond formation to form pre-translocation complexes were included in the subsequent analyses.
Replication	Replication and reproducibility were measured from the analysis of a sample size of 3. All attempts of replication were successful.
Randomization	Randomization is not relevant to this study, because all samples were designed to test a hypothesis and were compared to control samples where key components of the hypothesis were maintained constant.
Blinding	Blinding was not applicable to this study.

Reporting for specific materials, systems and methods

We require information from authors about some types of materials, experimental systems and methods used in many studies. Here, indicate whether each material, system or method listed is relevant to your study. If you are not sure if a list item applies to your research, read the appropriate section before selecting a response.

Materials & experimental systems

n/a	Involved in the study
[ ]	[x] Antibodies
[x]	[ ] Eukaryotic cell lines
[x]	[ ] Palaeontology and archaeology
[x]	[ ] Animals and other organisms
[x]	[ ] Human research participants
[x]	[ ] Clinical data
[x]	[ ] Dual use research of concern

Methods

n/a	Involved in the study
[x]	[ ] ChIP-seq
[x]	[ ] Flow cytometry
[x]	[ ] MRI-based neuroimaging

Antibodies

Antibodies used	Rabbit polyclonal anti-lolB antibodies (generated by Tokuda and provided as a gift, https://pubmed.ncbi.nlm.nih.gov/9384574/), used at a 10,000 dilution;<br>Rabbit polyclonal anti-CysRS antibodies (generated in the Hou lab, and published in Masuda et al., 2019, https://pubmed.ncbi.nlm.nih.gov/30981730/), used at a 20,000 dilution;<br>Goat polyclonal anti-rabbit IgG antibodies peroxidase conjugate (Sigma-Aldrich, Cat #A0545).
Validation	Rabbit polyclonal anti-lolB antibodies were validated by recognition of purified monomer of E. coli LolB in Western blot analysis (https://pubmed.ncbi.nlm.nih.gov/9384574/).<br>Rabbit polyclonal anti-cysRS antibodies were validated by recognition of purified E. coli CysRS in Western blot analysis (pubmed.ncbi.nlm.nih.gov/30981730/).
